# Conservative non-pharmacological interventions in women with pelvic floor dysfunction: a systematic review of qualitative studies

**DOI:** 10.1186/s12905-022-02097-y

**Published:** 2022-12-12

**Authors:** Ana Carolina Nociti Lopes Fernandes, Domingo Palacios-Ceña, Caroline Caetano Pena, Thaiana Bezerra Duarte, Aura Maria Paternina de la Ossa, Cristine Homsi Jorge

**Affiliations:** 1 Ribeirão Preto Medical School, University of São Paulo, Av. Bandeirantes, 3900, Monte Alegre, Ribeirão Preto, SP CEP: 14049-900 Brazil; 2 University Rey Juan Carlos, Av. Atenas s/n, CP: 28922 Alcorcón, Madrid, Spain; 3 University Center of Northern, Av. Joaquim Nabuco, 1232, Centro, Manaus, AM CEP: 69020-031 Brazil

**Keywords:** Physiotherapy, Pelvic floor, Women’s health, Women’s experience

## Abstract

**Background:**

Women’s adherence is essential to physiotherapeutic treatment of pelvic floor dysfunction, but its related factors are not usually considered in the development of treatment approaches. This study aims to understand how women with pelvic floor dysfunction experience pelvic floor conservative non-pharmacological treatment options.

**Methods:**

A systematic review of qualitative studies. The electronic search was performed in MEDLINE/PubMed, CINAHL, Lilacs, SCOPUS, and Web of Science databases. Primary articles on qualitative methods focused on the experience of women regarding pelvic floor conservative non-pharmacological interventions, i.e., pelvic floor muscle training (PFMT), either associated or not with biofeedback, perineal massage, vaginal dilators, and others. A meta-aggregation was performed.

**Results:**

It was included 22 manuscripts in this review. It was found seven studies about the use of vaginal devices, two about manual intervention and 14 studies on women’s experience with PFMT. The findings were synthesized as follows: I) women’s experience of manual interventions; II) women’s experience using vaginal devices changes according to health professional attitudes; III) women’s experiences using vaginal devices varied depending on their pelvic floor dysfunction; IV) reported side effects due to the use of vaginal devices; V) external factors influencing PFMT performance; VI) women’s perception of their own personal factors influencing PFMT performance; VII) PFMT characteristics influencing women’s adherence; VIII) strategies used by women to include PFMT in their routine.

**Conclusion:**

Women’s experience with pelvic floor conservative non-pharmacological treatment options is a complex phenomenon that involves many more variables than simply personal aspects. This is a systematic review of qualitative studies registered in the PROSPERO (CRD42018080244).

## Background

Pelvic floor dysfunction (PFD) is a term used to describe any disturbance in the active (i.e., pelvic floor muscle) and/or passive (i.e., fascia and ligaments) components of the pelvic floor [1]. In the current literature, pelvic floor muscle training (PFMT) is known to be the gold standard treatment for PFD, specifically for urinary incontinence (UI) and pelvic organ prolapse (POP) in women [2, 3]. Other conservative non-pharmacological treatment options for PFD include electrical nerve stimulation, perineal massage, vaginal dilators and pessaries [4, 5].

Women’s adherence is an essential component to be considered during conservative non-pharmacological treatments for PFD [6]. Adherence and its related factors are not usually considered in the development of different treatment approaches, nor as a primary outcome measure in randomized controlled trials (RCT), which is the appropriate study design to determine the effectiveness of any pelvic floor conservative non-pharmacological interventions [7–9].

A recognized limitation of RCTs is they do not allow an understanding of women’s experience with the intervention under investigation, nor aspects that may influence their adherence to treatment [9–11]. Thus, qualitative research aiming to understand the experience of women with conservative non-pharmacological treatments for PFD can fill this important gap [10]. To date, no specific systematic reviews about this important topic was found.

Thus, the aim of this study was to perform a systematic review of qualitative studies to answer the following question: how do women with PFD symptoms experience conservative non-pharmacological treatment options?

## Methods

This is a systematic review of qualitative studies registered in the PROSPERO (CRD42018080244) which search was conducted in April 2020. The research was planned based on SPIDER: I) Sample—women with PFD symptoms; II) Phenomenon of Interest—conservative non-pharmacological treatment options (PFMT, vaginal dilators, biofeedback, perineal massage, pessary, and others); III) Design—qualitative research, thematic analysis, grounded theory, phenomenology; IV) Evaluation—women’s experience; V) Research type—qualitative studies.

The inclusion criterion was original qualitative research regarding women’s experience with pelvic floor non-pharmacological interventions. The exclusion criteria were: (1) not primary research; (2) quantitative or mixed methods studies; (3) studies about women’s experience with the treatment of their partners or children; (4) studies that included either only men or both men and women. Although systematic reviews, quantitative and mixed methods studies were not included in this review, their references were examined to identify any additional study that meet the inclusion criterion.

The electronic search was conducted by one researcher (ACNLF). No limit was set for year of publication. The last search was performed in April 2020. The primary research was conducted in MEDLINE/PubMed, CINAHL, SCOPUS, Lilacs and Web of Science databases, and is summarised in Table 1.

**Table 1:** Search strategy

Database	SPIDER	Strategy
MEDLINE/PubMed CINAHL SCOPUS Web of Science	Sample	(“pelvic floor dysfunctions” OR “lower urinary tract symptoms” OR “urinary incontinence” OR “anal incontinence” OR constipation OR “fecal incontinence” OR “pelvic organ prolapse” OR “sexual dysfunction” OR vaginismus OR dyspareunia OR “pelvic floor”)
	Phenomenon of Interest	(physiotherap* OR “pelvic floor muscle training” OR “pelvic floor exercise” OR “Kegel exercise” OR “behavioral treatment” OR “behavioral therapy” OR “perineal massage” OR “vaginal dilators” OR “vaginal cones” OR biofeedback OR “electrical nerve stimulation” OR “electrical stimulation” OR pessaries OR “exercise therapy” OR treatment OR therap*)
	Design	(“qualitative research” OR “qualitative study” OR qualitative OR “thematic analysis” OR “grounded theory” OR phenomenology OR “focus group” OR “semi-structured interview”)
	Evaluation	–
	Research type	–
Lilacs	Sample	(“lower urinary tract symptoms” OR incontinenc$ OR constipation OR constipação OR “pelvic organ prolapse” OR “prolapso dos órgãos pélvicos” OR “sexual dysfunction” OR "disfunção sexual" OR vaginism$ OR d?spareunia OR “pelvic floor” OR "assoalho pelvico")
	Phenomenon of Interest	(physiotherap$ OR fisioterapia OR treinamento OR exercicio$ OR tratamento OR massagem OR dilatador$ OR cone$ OR “estimulação elétrica” OR “electrical stimulation” OR training OR exercise OR treatment$ OR massage OR dilators OR biofeedback OR pessari$)
	Design	–
	Evaluation	–
	Research type	qualitative

The search result was imported to the EndNote online platform where duplicates were excluded. The remaining references were first selected according to the relevance of their title and abstract to the research question. The selection was conducted by two independent researchers (ACNLF and TBD) who carefully read the full texts. Any disagreement was solved by discussion with a third reviewer (DPC). A secondary search was conducted manually using the selected articles and the reviews and mixed-method studies found during manual searching.

One researcher (CCP) was responsible for extracting the following information: bibliographic details, population, setting, cultural information, aims of the study, specific qualitative methodology, sampling method and size, and main results. Data synthesis was conducted after data extraction and fragments of participant reports was used to support the finding of this review. A second researcher (ACNLF) checked the extracted information as well as the congruence between the findings and the text fragments used. Divergences on selected information were settled through discussion between researchers.

The quality of the selected studies was assessed based on the quality criteria for qualitative studies, the Critical Appraisal Skills Programme checklist. This 10-question checklist covers three broad issues, named: are the results of the study valid (Section A—questions 1 to 6)? What are the results (Section B—questions 7 to 9)? Will the results help locally (Section C—question 10)?

A meta-aggregation [12] was conducted as follows: (1) extraction of all findings (including narrative fragments and quotes); (2) developing categories; (3) developing synthesised findings. Findings and categories were grouped based on similarity of concept and no software was used.

## Results

**Fig. 1:**
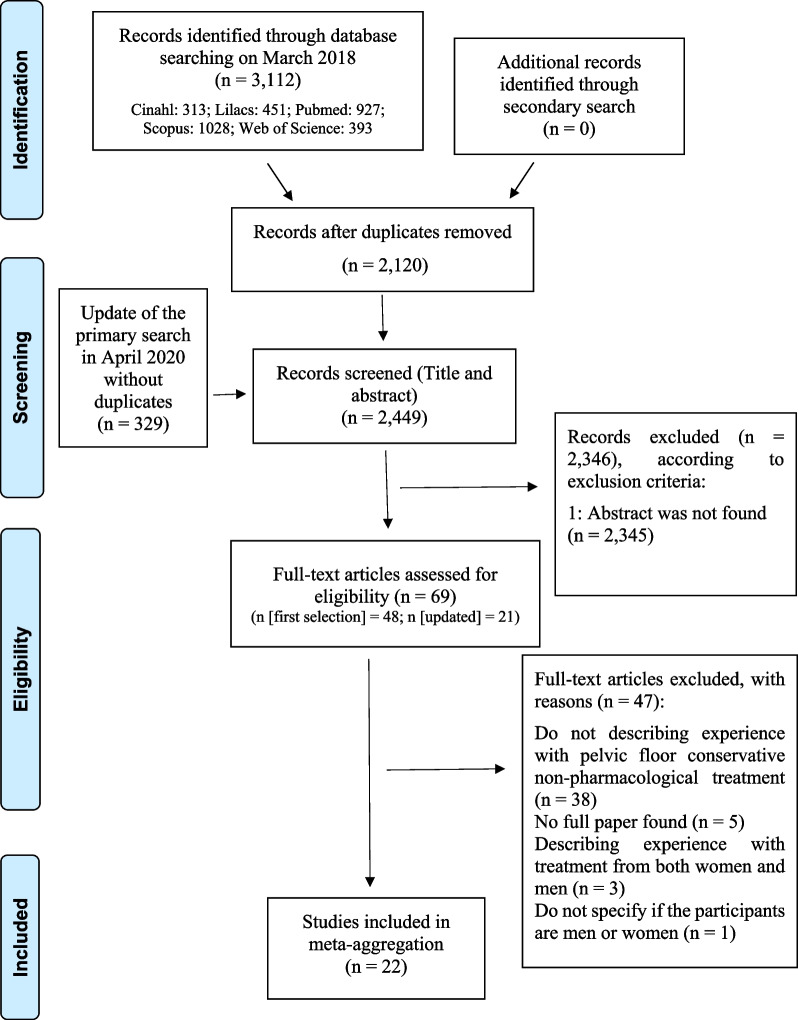
Study selection flow diagram

The flowchart of the study is presented in Fig. 1. It included 22 manuscripts published between 1993 and 2020, with a total of 304 participants.

### Study characteristics

Table 4 in Appendix presents the characteristics of the included studies. The studies were conducted in different parts of the world: two (9.1%) studies were from South America [13, 14]; four (18.2%) from North America [15–18]; ten (45.4%) from Europe [19–28]; two (9.1%) from Asia [29, 30] and three (13.6%) from Oceania [31–33]. One study (4.5%) did not report where data collection was performed [34].

From the 22 manuscripts included, 13 (59%) aimed to understand women’s experience with PFMT [13, 14, 20, 22–24, 27–30, 32–34], three (13.6%) with vaginal dilators [21, 25, 31], three (13.6%) with pessaries [16–18] and one (4.5%) with perineal massage [26]. One (4.5%) study aimed to understand women’s experience using hands-on physiotherapeutic interventions treating sexual dysfunction [15], and another one (4.5%) investigated women’s experience with pessaries and PFMT use [19].

#### Synthesis of the results

The synthesis of qualitative results is presented as follows: I) women’s experience of manual interventions; II) women’s experience using vaginal devices changes according to health professional attitudes; III) women’s experiences using vaginal devices varied depending on their pelvic floor dysfunction; IV) reported side effects due to the use of vaginal devices; V) external factors influencing PFMT performance; VI) women’s perception of their own personal factors influencing PFMT performance; VII) PFMT characteristics influencing women’s adherence; VIII) strategies used by women to include PFMT in their routine. Narrative fragments of the findings are presented in Table 2.

**Table 2:** Synthesised findings and narrative fragments regarding women’s experience with vaginal devices, manual interventions and PFMT

Treatment category	Synthesised findings	Narrative fragments
Manual interventions	Women’s experience of manual interventions	Q.1: “Um, obviously it did feel as though someone was right inside so, when they were pushing down on it, obviously, it hurt. But, after, like, I think it was her thumb or her finger that had been there for ages, it would release off and then you couldn’t feel anything.” [26] Q.2: “…It feels loads better now. I’ve only been, like, three, about three or four times, but it just feels loads different already.” [26] Q.3: “It was helpful because it was very educational how much your muscles will tense up as a result of painful sex or actual pain […] You could see the activity of the muscles, [what] the tone of your muscles was, so if they were tight, you could see that on the screen and then see when you’re relaxing them […] you see it happening and so as a result you’re in better control of it.” [15]
Vaginal devices	Women’s experience using vaginal devices changes according to health professionals’ attitudes	Q.4: “Here they’ve given me information. How to put it on, how to take it off, and everything. I felt better.” [16] Q.5: "They sent me to see this lady to fit some sort of contraption, and she brought out this dice which was about two inches square…block on a string, and she spent 15 min writhing and tugging and trying to get it there, gave up and said, do it yourself. So I had a go, and I said, there’s no way that is going to fit in there. Well, she said, you’ve had two babies. I said, yes, I have, I said, but your bones soften and everything’s different, your physiology’s different when you’re having babies. I said, that’s not going to go in there, and if it does go in there, it ain’t going to come out, so I'm not using it.” [19] Q.6: “You lie down in a specific position that makes you feel quite vulnerable, and there is a doctor standing at the opposite end of you and she is trying to stick her finger in you [both laugh]. And then it doesn't go in and she is saying 'Relax. Relax. Relax.'” [25]
	Women reported varied experiences using vaginal devices depending on their pelvic floor dysfunction	Q.7: “I just looked at it as a furthering of the treatment basically.” [31] Q.8: “The idea repulsed me a little. Well maybe because, just after that operation and brachytherapy and such, the medical world frequently inserted all kinds of objects [vaginally], and that made you feel like: not now.” [21] Q.9: “I did feel a little scared of having this [pessary] inside of me…I thought I was going to feel more uncomfortable than I was already feeling. But no, I eventually got accustomed to it.” [16] Q.10: “What do I like about my pessary? Well, it gives me more confidence that I can go out and be active. You know, it’s bad always trying to be close to a washroom wherever I go. I’m very comfortable using it, it gives me some security that I can go out and enjoy myself, either to a party, or to our lunches or play darts or whatever.” [17]
	Side effects while using vaginal devices	Q.11: “But it was also kind of scary [referring to blood loss during dilator use] (…)”[21] Q.12: “The only thing that I’d change is that I had some bleeding when I first used the dilator. It would have been nice if someone had warned me about this.” [31] Q.13: “Well, I perform... Yes, it’s weird, but I perform dilator use while listening to music from Wagner. Because it is a music piece that lasts exactly 10 min.” [21] Q.14: “Those things [referring to dilators] aren’t enjoyable sex toys. [...] Then I think: then you also have to pay for them as well. [...] I also let them know that I found that very disturbing” [21]
PFMT	External factors influencing PFMT performance	Q.15: “My family encourages me to do pelvic floor muscle exercise. Sometimes while I am sitting and watching television, my daughter will remind me to do it. She says, “You should work harder, otherwise it will not be so good”. They are very supportive.” [30] Q.16: “You showed me exactly what to do, and went through it, and made it easier- I thought well I’m not so [uncoordinated] as I thought.” [32] Q.17: “I was given a leaflet, ehm nobody really demonstrated or kind of showed you how to do the pelvic floor exercises." [23]
	Women´s perception of their own personal factors influencing PFMT performance	Q.18: “I think being in control it just sort of contributes to your well-being.” [33] Q.19: “Pelvic floor muscle exercises are pretty tough. […] It’s easy to pick the wrong muscles although you might be thinking you’re doing well. It would be nice to have an expert to check it; in the computer cannot do that.” [27] Q.20: "For me because I don’t have a lot of confidence in my own abilities I would probably want somebody else to check.” [28] Q.21: "I quite enjoy being quite competitive with myself and trying to achieve wee goals and being on my own with exercise." (about an app) [23] Q.22: “In the beginning, when you asked me to contract there (vaginal muscle), I associated with that thing (sex). When I twitched and contracted there (the vagina) I felt embarrassed.” [29] Q.23: “It’s a bit tough, I think, difficult. I don’t know if I’m doing it right.” [24] Q.24: “I never tried to squeeze underneath (the vagina) before. I remember when you taught me to contract there (vagina), my first thought was: Could they be contracted? I didn’t even know where they were. I couldn’t feel them, because they could not be touched and also, you are not touching them.” [29] Q.25: "No, it’s more that I’ve tested it out and feel that I’m becoming successively stronger and stronger, so I have gotten that confirmation, that this was something important. Of course, the support in the instructions, what I would experience, that I would feel it dropping, feel it relaxing, and that’s exactly what I felt." [20]
	PFMT characteristics influence women’s adherence	Q.26: “It [PFMT] was different, because it wasn’t what you would call real exercise, I know that’s silly, ‘cause it was, just a different area, and not everybody can see you doing it. The silent, private exercise.” [32]
	Strategies used by women to include PFMT in their routine	Q.27: “Yeah yeah so the last thing I’ve been thinking about (laughter) is my pelvic floor exercises.” [33] Q.28: “I have a habit of touching my ear lobe each morning to remind myself of the things that I have to do… each day when I touch my earlobe, I will remember to do the exercise. I absolutely will keep on with it.” [30]

*PFMT* pelvic floor muscle training; *Q* quotes

### Synthesised findings I: Women’s experience of manual interventions

Women undergoing physiotherapeutic treatment involving perineal massage to treat painful sexual complaints reported its deep impact on a physical, emotional and social level [15, 26]. They reported health professionals' attention as careful, and they felt that their complaints were taken seriously. The treatment makes them have a better perception of their body sensations, as well as the feeling of great relief while muscle spasms decreased (Table 2—Q.1, Q.3). All women reported feeling “better and better” after treatment.

### Synthesised findings II: Women’s experience using vaginal devices changes according to health professional attitudes

The studies presented the experience of women who had undergone pelvic radiotherapy [21, 31], as well as women diagnosed with vaginismus [25], POP or UI [16–19]. Although there are differences in the studied sample, a common point needs to be highlighted: the communication between health professional and patient. The communication aspect of the treatment seems to have a negative influence on women’s experience using vaginal dilators, while it has a more positive impact during the use of pessaries (Table 2—Q.4).

Women seems to become insecure using vaginal dilators [21, 25, 31] due to conflicting information offered by health professionals with different levels of communication skills, however, some women described that health professional support was essential to providing a good experience during treatment [16–19]. Poor interaction between health professionals and patients was reported as a reason for discontinuation of treatment (Table 2—Q.5).

### Synthesised findings III: Women’s experiences using vaginal devices varied depending on their pelvic floor dysfunction

For some women, using vaginal devices was simply an aspect of their treatment to reduce discomfort with their symptoms (Table 2—Q.7), while others perceived the use of a vaginal dilator as a constant confrontation of the reality of cancer (Table 2—Q.8). Only those using pessaries reported having an active role in the process of choosing or not choosing this treatment option. Reasoning for not choosing this treatment was based on other women’s experiences.

Despite some women with vaginismus reported a positive experience, most of them described it as “painful”, “humiliating” and even “traumatic”. The smallest vaginal dilator was considered too big, leading women to seek alternatives (e.g., vibrators and tampons). The transition between devices was difficult due to the jump in size. They stated that their journey would be easier if they cold count with partner, professional and peer support. In contrast, women using pessaries reported needing some time to learn how to use pessaries in an effective way, and once they learned how to manage it they perceived the treatment as a life-changing experience, using words such as “freedom”, “security” and “satisfaction” (Table 2—Q.9, Q.10).

### Synthesised findings IV: Reported side effects due to the use of vaginal devices

Only studies on vaginal dilators presented reports of side effects, including blood loss, pain and vaginal discharge (Table 2—Q.11, Q.12). These negative experiences seem to result in anxiety, but also as a motivation to continue using dilators. Reported strategies to reduce negative feelings were increasing positivity and integrating the treatment into daily life, such as during a bath (Table 2—Q.13). The sexuality involved in the use of the vaginal dilator was a controversial experience (Table 2—Q.14).

### Synthesised findings V: External factors influencing PFMT performance

The extrinsic factors appearing to influence PFMT performance are health care professional and family support (Table 2—Q.15). Health professionals were considered possible facilitators or barriers to PFMT performance (Table 2—Q.16). For some women their complaints were not properly accept by some health professionals, and they felt the need for further instructions to be able to perform PFMT. These women complained that when they received the information it was not in an appropriate manner (Table 2—Q.17). However, when the information and support were given properly, it helped them in adhering to PFMT.

### Synthesised findings VI: Women’s perception of their own personal factors influencing PFMT performance

This theme has three subthemes:

*Category I: Positive personal factors*. A positive experience for women undergoing PFMT was reported as the feeling of control over their body (Table 2—Q.18). To avoid incorrect training, the ability to perceive and confirm their capacity to contract their PFM was considered important by women, in order to improve their commitment and confidence in their own ability to perform PFMT (Table 2—Q.19, Q.20) and to diminish their symptoms. A participant mentioned putting her own competitiveness in playing mobile apps games as a positive factor to perform PFMT (Table 2—Q.21).

*Category II: Negative personal factors.* Women mentioned the following negative factors: lack of motivation; previous negative experience with PFMT; uncertainties about the results or lack of good results; limited understanding about how PFMT works; embarrassments or conflicting feelings about erotization (Table 2—Q.22); difficulties in PFMT performance, including the “invisibility” of the muscle; uncertainty whether or not they were correctly contracting their PFM (Table 2—Q.23); emotional, mood and climactic factors; guilty for not performing PFMT; worsening of the symptoms; loneliness while performing PFMT; perception that PFMT is boring or a waste of time; and the naturalisation of PFD symptoms.

*Category III: Perineal perception*. While some women reported an inability to perceive a correct PFM contraction, some highlighted that they could progressively perceive it and notice whether or not they were performing it correctly (Table 2—Q.24, Q.25).

### Synthesised findings VII: PFMT characteristics influence women’s adherence

Some women considered they have a better adherence to PFMT performed in groups, while others classified PFMT as a quiet, “private exercise” (Table 2—Q.26)*.* While for some women PFMT could be done at any time of day without anyone else knowing, others emphasized their need to have a quiet place to focus on it. A positive point highlighted was the association of PFMT with other benefits, such as improving their self-confidence. The commitment involved in becoming a participant in clinical studies was perceived as one’s own obligation to adhere to treatment. It is worth noting that PFMT was not seen as a “real exercise” by some participants (Table 2—Q.26). This deconstruction of PFMT as physical exercise was justified by the anatomical region of the PFM, and by the possibility of performing contractions during penetrative vaginal sex.

### Synthesised findings VIII: Strategies used by women to include PFMT in their routine

Some participants justified their lack of adherence to PFMT due to the lack of time to perform it. Other participants were able to include PFMT in their routine by associating it with daily life activities, such as performing the exercise while waiting for the bus or doing it every time they sit in their computer chair, among other situations (Table 2—Q.28).

#### Quality criteria assessment

Table 4 shows that only four studies reported the use of any form of quality criterion and only one manuscript reported the use of the Consolidate Criteria for Reporting Qualitative Research (COREQ).

Table 3 presents the studies’ results of quality criteria analysis. Only six (26.1%) articles received the maximum score of section A, and 21 (91.3%) articles were considered to have clear (section B) or relevant results (section C). The quality of most included manuscripts was limited in aspects of methodology. There was a high number of manuscripts that did not present clear information about the recruitment strategies, nor regarding the relationship between the researchers and the participants.

**Table 3:** Quality criteria

Author	Clear statement of the aims of research	Appropriateness of qualitative design	Research design consistent to address the aims of research		Recruitment strategies appropriate to the aims	Data collection strategy appropriate	Relationship considered between research and participants	Ethical issues considered	Data analysis rigorous	Clear statement of the results	How valuable is the research?
Ashworth et al. [34]	X	✓		✓	✓	✓	✓	X	✓	✓	–
Bakker et al. [21]	✓	✓		✓	✓	✓	✓	✓	✓	✓	✓
Bjork et al. [22]	✓	✓		✓	✓	✓	✓	✓	✓	✓	✓
Bonner et al. [31]	✓	✓		✓	✓	✓	–	✓	✓	✓	✓
Cacchioni et al. [15]	X	–		–	–	–	–	–	–	✓	–
Delarmelindo et al. [13]	✓	✓		✓	–	✓	–	✓	✓	✓	✓
Delarmelindo et al. [14]	✓	✓		✓	–	✓	–	✓	✓	✓	✓
Firet et al. [27]	✓	✓		✓	✓	✓	✓	✓	✓	✓	✓
Hay-Smith et al. [32]	✓	✓		✓	✓	✓	–	✓	✓	✓	✓
Hayland et al. [33]	✓	✓		✓	–	✓	–	✓	✓	✓	✓
Kao et al. [29]	✓	✓		✓	✓	✓	–	✓	✓	✓	✓
Lindgren et al. [24]	✓	✓		✓	✓	✓	–	✓	✓	✓	✓
Macey et al. [25]	✓	✓		✓	✓	✓	–	✓	✓	✓	✓
Mackenzie [26]	✓	✓		✓	✓	–	✓	✓	✓	✓	✓
Sevilla et al. [16]	✓	✓		✓	✓	✓	✓	✓	✓	✓	✓
Siu et al. [30]	✓	✓		✓	–	✓	–	✓	✓	✓	✓
Storey et al. [17]	✓	✓		✓	✓	✓	–	✓	✓	✓	✓
Firet et al. [27]	✓	✓		✓	✓	✓	✓	✓	✓	✓	✓
Maldonado et al. [18]	✓	✓		✓	✓	–	–	✓	✓	✓	✓
Asklund et al. [20]	✓	✓		✓	✓	✓	✓	✓	✓	✓	✓
Grant et al. [23]	✓	✓		✓	✓		–	✓	✓	✓	✓
Abhyankar et al. [19]	✓	✓		✓	✓	✓	–	✓	✓	✓	✓
Terry et al. [28]	✓	–		✓	✓	–	–	✓	✓	✓	✓

Symbols indicate: X = No; – = Can't tell; ✓ = yes

## Discussion

This study aimed to understand how women with PFD experienced pelvic floor conservative non-pharmacological treatment options. We included studies reporting women’s experience with different conservative non-pharmacological options to treat PFD.

The experience of women with PFMT seems to be related to several personal factors. The understanding by physiotherapists of factors modulating the quality of women’s experiences with this intervention seems to be essential to improve it. Although the large amount of scientific evidence showing PFMT as a treatment for some PFD symptoms is well-established in the literature, adherence remains the most challenging aspect of this treatment [2, 3, 9, 35]. PFMT adherence is a complex phenomenon that involves the active participation of patients. This study reinforces the need of women to receive further appropriate information to modify their behaviour, incorporating PFMT practice in their routine [9, 35]. This perception is aligned with the results of studies showing women’s general lack of knowledge related to PFM function, dysfunction and options of treatment, including PFMT [36]. Women with different background can acquire basic knowledge about PFMT after receiving information about the pelvic floor location/anatomy and PFM function [37]. Other studies have indicated that when women receive information about the pelvic floor they have a higher chance of adherence to PFD conservative interventions [6, 8]. Still, many women consider they don’t receive information based on their specific background and needs.

Another important aspect to be considered is women’s belief in their ability to perform PFMT, commonly known as self-efficacy. This belief is the core of social cognitive theory, one of the many theories and methods described in the literature that can be used as a guide while working with health behaviour [6]. The use of a more patient-centred approach may improve not only self-efficacy but other personal factors as technical abilities that deeply influence women’s experience with PFMT. Additionally, women referred to their ability to perceive or not perceive their PFM contraction, respectively, as a facilitator or barrier to PFMT. Self-perception as a modifier of PFMT adherence is an aspect which could be considered and worked on, as one study shows that women’s estimation of their PFM contraction intensity is poor, especially in women with a non-contracting or a weak PFM [38]. An increase in women’s perception of their PFM contraction seems to be another positive result of PFMT that could be further explored using a self-efficacy approach.

Similarly, to the strategies suggested to improve PFMT adherence, health behavioural theories have been used to identify and fill knowledge gaps related to continence promotion [39, 40]. The study conducted by Chiarelli and Cockburn [39] identified, through focus groups, gaps related to women’s knowledge after delivery and, using Health Belief Model as a theoretical guide, proposed an education program aiming to promote urinary continence (UI). To verify the program’s effectiveness, a RCT was conducted and concluded that the intervention group showed reduced prevalence of UI with adequate levels of PFMT adherence, compared to the standard care procedure group three months after childbirth [40]. It is important to highlight that these are the few studies in women’s health physiotherapy which used this approach and no studies were found specifically for the use of vaginal devices. Unfortunately, there is a small number of studies using this approach to improve women’s adherence to other interventions such as vaginal dilators.

This systematic review found reports of some negative experiences of women using vaginal dilators. However, women’s reports of intentionally associating the use of vaginal dilators with pleasant situations was identified as an important coping strategy that improved their experience.

The experiences reported after the use of pessaries were varied, but they underline the importance of women’s participation in the process of choosing the intervention, and the essential role of the health professional in either continuity or interruption of the treatment. In the only two studies investigating pelvic floor manual interventions, women stated having had a good experience while using it, especially due to the support given by the health professional.

We must state the limitation of this systematic review reflects the limitation of the included studies. Most of the included studies had methodological shortcomes and none of those articles were excluded from the review. Nevertheless, this review brings together the experiences of women with a variety of conservative interventions, highlighting important aspects that may contribute to better healthcare assistance related to PFD and to improve both treatment adherence and satisfaction.

The results showed relevant aspects that should be considered during treatment approach (e.g., adequate communication, adequate provision of information, and appropriate support from health professionals), to particularly improve women’s experience and adherence to the interventions.

This review also uncovered the need for more qualitative studies with a strong methodology to better understand women’s experience with pelvic floor conservative non-pharmacological interventions, especially those regarding manual interventions and vaginal devices.

## Conclusion

Women’s experience with pelvic floor conservative non-pharmacological treatment options is a complex phenomenon that involves many more variables than just personal aspects. A more patient-centred approach should be considered to improve women’s experience with and adherence to conservative options.

## Data Availability

The datasets used and/or analysed during the current study are available from the corresponding author on reasonable request.

## Appendix

See Table 4.

**Table 4:** Characteristics of selected studies

Identification	Qualitative design	Sampling strategies	Number/ participants	Intervention	Tools	Analysis	Quality criteria reported
Abhyankar et al. [19]	Qualitative study—symbolic interactionism	Purposive sampling	22 women with POP receiving prolapse care	PFMT and/or pessaries	Semi-structured interviews and focus group	Thematic analysis	–
Ashworth et al. [34]	Phenomenological study	Unknown	28 women with UI and aged between 25 to 55 years old	PFMT	Non-structured and structured face-to-face interview	Analysis was guided by phenomenological principles, adapting from Giorgi	–
Asklund et al. [20]	Qualitative study	Purposive sampling	15 women with SUI and aged between 27 to 72 years old (mean age 47 years old)	PFMT	Semi-structured, telephone interview, guide with open-ended questions	Grounded theory	–
Bakker et al. [21]	Qualitative study	Purposive sampling	30 women who treaded with brachytherapy (mean age was 49 years old, aged between 32 to 67 years old)	Vaginal dilator	Semi-structured face-to- face or by telephone interview	The transcriptions were analysed with QSR International's Nvivo 10 software using the Framework Approach	–
Bjork et al. 2014	Qualitative study	Strategic selection	21 women with SUI aged between 30 to 69 years old	PFMT	Semi-structured interview by telephone	Grounded theory	–
Bonner et al. [31]	Qualitative study	Purposive sampling	15 women with an average age was 53 years old, aged between 31 to 68 years old	Vaginal dilators	Semi-structured face-to-face or by telephone interview	Thematic analysis	–
Cacchioni et al. [15]	Qualitative study	Unknown	31 women were interviewed but only 18 of those interviewed were presented (average age of was 36 years old, aged between 24 to 62 years old)	Hands-on intervention	Semi-structured, in-depth interviews	All interview and fieldwork data were transcribed and manually coded by theme with names and identity-revealing information changed	–
Delamerlindo et al. [13]	Grounded theory study	Purposive sampling	18 women aged between 41 to 81 years old	PFMT	Non-directive interview	Grounded theory	–
Delamerlindo et al. [14]	Qualitative study	Unknown	18 women with UI aged between 41 to 81 years old	PFMT	Non-directive interview	Grounded theory	–
Firet et al. [27]	Qualitative study	Purposive sampling	13 women with SUI aged between 40 to more than 80 years old	PFMT	Semi-structured, face-to-face interviews	Grounded theory and Atlas.ti software	The Consolidated Criteria for Reporting Qualitative Research
Grant et al. [23]	Qualitative exploratory study	Unknown	31 postnatal women aged between 28 to 43 years old	PFMT	Online and face-to-face focus group	Thematic analysis	–
Hay-Smith et al. [32]	Unknown	Purposive sampling	20 women with aged between 23 to 86 years old	PFMT	Semi-structured face-to-face interviews	Descriptive content analysis	–
Hyland et al. 2013	Phenomenological study	Purposive sampling	5 women with POP aged between 45 to 60 years old	PFMT	Semi-structured face-to-face interviews	Interpretative phenomenological analysis	–
Kao et al. [29]	Qualitative descriptive study	Purposive sampling	12 women aged between 44 to 66 years old	PFMT	Semi-structured face-to-face in-depth interviews	Thematic analysis	Lincoln and Guba's guidelines: Credibility, Dependability, Confirmability and Transferability
Lindgren et al. [24]	Qualitative descriptive study	Unknown	13 women who survived gynaecological cancer, mean age of 66 years old (aged between 48 to 82 years old)	PFMT	Semi-structured interview	Content analysis	–
Macey et al. [25]	Qualitative study—phenomenological approach	Convenience sampling	13 women were interviewed aged between 20 to 67 years old	Vaginal dilators	Semi-structured interviews	Thematic analysis	Although the article did not present in writing the use of quality criteria, some could be identified during the reading
Mackenzie [26]	Phenomenological study	Phenomenological sampling	5 women	Perineal massage	Semi-structured, in-depth interviews	Colaizzi's data analysis framework	Verbatim quotes are utilised in reports of the findings to further enhance credibility, subjects were only invited to participate if they had had no prior contact with the researcher
Maldonado et al. [18]	Qualitative study	Unknown	29 Spanish-speaking women with symptoms of POP (aged between 40 to 79 years old, mean 50)	Pessaries	Semi-structured interview/in focus group	Grounded theory	–
Sevilla et al. [16]	Qualitative study	Unknown	16 Spanish-speaking women with a mean age of 67.6 (aged between 47 to 85) years old	Pessary	Semi-structured interviews	Grounded theory	–
Siu et al. [30]	Qualitative descriptive study	Purposive sample	35 women with SUI who aged between 39 to 74 years old with a mean age of 51.26	PFMT	Semi-structured face-to-face interviews	Content analysis	The rigour in qualitative research followed the techniques suggested by Tuckett
Storey et al. [17]	Interpretative narrative inquiry	Purposive sampling	11 women with UI and/or POP who were over 65 years old and retired and came from both rural and urban areas	Pessaries	Semi-structured face-to-face interviews	Narrative analysis	Trustworthiness, an audit trail of researcher field notes and reflexive texts, documenting decision-making, biases and analytical choices
Terry et al. [28]	Ethnographic approach	Unknown	15 pregnant women and 12 of these women postnatally	PFMT	Observations, interviews, field conversations, comprehensive field notes and semi-structured interviews	Thematic analysis	

*SUI* Stress Urinary Incontinence; *UI* Urinary Incontinence; *PFMT* Pelvic Floor Muscle Training; *US* United States; *POP* Pelvic Organ Prolapse

## Abbreviations

PFMT: Pelvic floor muscle training
PFD: Pelvic floor dysfunction
RCT: Randomised controlled trial
UI: Urinary incontinence
POP: Pelvic organ prolapse
